# Potassium-competitive Acid Blockers: Current Clinical Use and Future Developments

**DOI:** 10.1007/s11894-024-00939-3

**Published:** 2024-08-15

**Authors:** Carmelo Scarpignato, Richard H. Hunt

**Affiliations:** 1 Department of Medicine & Surgery, University of Parma, Parma, Italy; 2 Department of Health Sciences, United Campus of Malta, Msida, Malta; 3 Faculty of Medicine, Chinese University of Hong Kong, Hong Kong, Hong Kong; 4 Faculty of Medicine, University of Nantes, Nantes, France; 5 Department of Medicine, Division of Gastroenterology and Farncombe Family Digestive, Health Research Institute, McMaster University, Hamilton, ON Canada

**Keywords:** GERD, *H. pylori* Infection, Unmet Clinical Needs, PPIs, P-CABs, Vonoprazan, Tegoprazan, Fexuprazan, Kerverprazan, Linaprazan glurate, Zestaprazan

## Abstract

**Purpose of the Review:**

Acid suppression with proton pump inhibitors (PPIs) represents the standard of care in the treatment of acid-related diseases. However, despite their effectiveness, PPIs display some intrinsic limitations, which underlie the unmet clinical needs that have been identified over the past decades. The aims of this review are to summarize the current status and future development of the new class of antisecretory drugs (potassium-competitive acid blockers, P-CABs) that have recently been introduced into medical practice.

**Recent Findings:**

Over the past decades, clinical needs unmet by the current acid suppressants have been recognized, especially in the management of patients with GERD, *Helicobacter pylori* infection and NSAID-related peptic ulcer. The failure to address these needs is mainly due to their inability to achieve a consistent acid suppression in all patients and, particularly, to control nighttime acidity. It was then realized that an extended duration of acid suppression would exert additional benefits. The available data with P-CABs show that they are able to address these unmet clinical needs.

**Summary:**

Four different P-CABs (vonoprazan, tegoprazan, fexuprazan and keverprazan) are currently available. However, only two of them are approved outside Asia. Vonoprazan is available in North, Central and South America while tegoprazan is marketed only in Latin American countries. Two other compounds (namely linazapran glurate and zestaprazan) are presently under clinical development. While clinical trials on GERD have been performed with all P-CABs, only vonoprazan and tegoprazan have been investigated as components of *Helicobacter pylori* eradication regimens. The available data show that—in the above two clinical indications—P-CABs provide similar or better efficacy in comparison with PPIs. Their safety in the short-term overlaps that of PPIs, but data from long-term treatment are needed.

## Introduction

The past century has been dominated by the Schwartz’ dictum “No acid, no ulcer” [1]. Indeed, the role of acid in the pathophysiology of gastric mucosal lesions represented the impetus guiding research into the treatment of acid-related diseases. Although antacids and anticholinergics were, until the 1970’s, the only medications available, albeit with limited efficacy, it was the discovery of H_2_-receptor antagonists (H_2_RAs) and proton pump inhibitors (PPIs) that brought a real breakthrough in the medical management of several acid-peptic conditions, relegating surgery to a very limited role [2]. Now, in the third millennium, acid suppression with delayed release PPIs (DR-PPIs) represents the cornerstone of medical treatment of GERD and its complications [3–5]. However, already 20 years ago it became evident that, despite their efficacy in a majority of patients, PPIs are far from the ideal antisecretory drugs [6, 7]. As a consequence, the search for more effective treatments started looking at new antisecretory drugs, novel formulations as well as fixed drug combinations [8, 9].

Over time, clinical needs unmet by the current acid suppressants have been recognized, especially in the management of patients with GERD, *Helicobacter pylori* infection and NSAID-related peptic ulcer [6, 10–12]. The failure to address these needs is mainly due to identifiable intrinsic limitations of PPIs, especially their inability to achieve a consistent acid suppression in all patients and, particularly, to control nighttime acidity. It was then realized that an extended duration of acid suppression would exert additional benefits [9, 13, 14]. A number of new drugs and/or drug classes have been developed during the past two decades, including PPIs displaying a half-life longer than that of current ones (the so called *third generation PPIs*, which however never reached the market) and immediate release or modified release formulations of some currently available compounds (*for review see* [15]). These new approaches provided a small incremental improvement in the pharmacological control of acid secretion, which was however not sufficient to achieve the degree of control of acidity needed in patients with more complex clinical problems [16].

The true innovation has been the introduction of the of H^+^,K^+^-ATPase blockers, called Potassium-Competitive Acid Blockers (P-CABs) [17–19], which share the same target as the PPIs (i.e. the gastric proton pump) but inhibit it through a completely different molecular mechanism. They block the K^+^ exchange channel of the proton pump, resulting in a very fast, competitive and reversible inhibition of acid secretion. Available P-CABs display a fast and longer-lasting elevation of intra-gastric pH than a DR-PPI. After the introduction in 2015 [20] of the first-in-class *successful* P-CAB (namely vonoprazan) into the Japanese market, other members of the class have been developed mainly in Asia, and brought also to North America and some Central/South American Countries.

The aims of this review are to summarize the relevant pharmacologic properties and clinical use of P-CABs (in particular those already approved and available for clinical practice) and show how they can, prescribed appropriately, address some of the current unmet clinical needs in acid-related diseases. In addition, those drugs under active clinical development will be discussed.

### P-CABs: Chemistry and Pharmacology

Conversely from the current PPIs, which are all substituted benzimidazoles, P-CABs belong to different chemical classes (Table 1). Despite acting through the same mechanism of action, they are therefore heterogeneous molecules. Being all lipophilic, weak bases with high pKa values and stable at low pH, P-CABs concentrate in acidic environments. For instance, a P-CAB displaying a pKa of 6.0 would theoretically have in the parietal cell canaliculus (pH = 1) a concentration 100.000-fold higher than in plasma (pH = 7.4), i.e., 1000 times more than a PPI [21].

**Table 1:** PK and PD characteristics of the currently available P-CABs (at the approved dose), given in fasting conditions, in the morning, to healthy *H. pylori*-negative Asian subjects

Drug (Regimen)	Revaprazan (200 mg daily)	Vonoprazan (20 mg daily)	Tegoprazan (50 mg daily)	Fexuprazan (40 mg daily)	Keverprazan (20 mg daily)
Structure	Pyrimidine	SulfonylPyrrole	Benzimidazole	SulfonylPyrrole	SulfonylPyrrole
Formula	C_22_H_23_FN_4_	C_17_H_16_FN_3_O_2_S	C_20_H_19_F_2_N_3_O_3_	C_19_ H_17_F_3_N_2_O_3_S	C_22_H_25_FN_2_O_4_S
M.W.	362.44	345.39	387.38	410.41	432.51
pKa	8.68	9.06	5.1	8.40	9.12
T_max_, h	2.1 ± 1.3	1.5 (1.5–3.0)	1.0 (0.5–2.0)	2.0 (1.5–4.0)	2.0 (1.2–2.0)
Half-life, h	2.4 ± 0.2	6.9 ± 1.6	4.1 ± 1.4	9.1 ± 1.2	6.3 ± 1.2
24 h pH, U—Day 1	2.2	NA	4.1 ± 0.7	3.6 ± 0.9	NA
24 h pH, U—Day 7	2.5	NA	4.7 ± 1.1	4.2 ± 0.9	NA
24 h pH > 4 Holding Time, %—Day 1	28.1	63.3 ± 17.9	54.5 ± 17.9	44.6 ± 22.9	85.0 ± 3.0
24 h pH > 4 Holding Time, %—Day 7	34.2	83.4 ± 16.7	68.2 ± 24.9	55.7 ± 19.2	98.3 ± 4.3
NAB Occurrence	Yes	No	No	No	No

All the studies concerning the different P-CABs (with the exception of kerveprazan) have been performed in Korean or Japanese subjects. Keverprazan was evaluated in healthy Chinese subjects. In the same study the effect of keverprazan was comparable to that of vonoprazan (24 h pH > 4 HTR: 99.4 ± 1.2 and 98.3 ± 4.3 for vonoprazan and keverprazan, respectively). NA = Not Available

The very first P-CABs that have reached clinical trials (i.e., SCH-28080, revaprazan and linaprazan) gave disappointing results. Development of SCH-28080 was stopped because of hepatotoxicity [22] while that of linaprazan (AZD0865) because of failure to show superiority over standard dose PPIs in healing reflux esophagitis [23], and detection of abnormal liver enzymes. However, although unable to achieve better healing of peptic ulcers [24, 25], revaprazan (YH1885) was introduced into the Korean and Indian markets. The development of other molecules (i.e., soraprazan, CS526 and YH-4808) were stopped after phase I or II because their administration was associated with transaminase elevation [26].

Conversely from earlier compounds, current P-CABs (either on the market or under clinical development) are devoid of hepatoxicity and display a more powerful and longer-acting inhibition of the proton pump compared with standard DR-PPIs. These drugs:

- Are stable in the acidic gastric environment, conversely from PPIs, which are acid-labile drugs. As a consequence, they do not need gastroprotection
- Display good solubility both in acidic and neutral conditions
- Accumulate into gastric mucosa and concentrate in the secretory canaliculi
- Exert a pH-independent and direct inhibitory activity on H^+^/K^+^-ATPase, without need for conversion into an active form. They are therefore not pro-drugs

The main pharmacologic differences between P-CABs and PPIs are summarized in Table 2**.**

**Table 2:** P-CABs and PPIs: Main Differences in the Mechanism of Action (*modified* from Scarpignato & Hunt [8])

P-CABs	PPIs
Acts directly (after protonation) on the H^+^,K^+^-ATPase enzyme	Requires transformation to the active form, sulphenamide
Super-concentrates in parietal cell acid space (100.000-fold higher than in plasma)	Concentrate in parietal cell acid space (1000-fold higher than in plasma)
P-CABs binds competitively to the K^+^ binding site of to H^+^,K^+^-ATPase	Sulphenamide binds covalently to H^+^,K^+^-ATPase
Reversible binding to the proton pump, *ionic*	Irreversible binding to the proton pump, *covalent*
Able to inhibit new proton pumps	Unable to inhibit new proton pumps
Duration of effect related to half-life of drug in plasma	Duration of effect related to half-life of the sulphenamide-enzyme complex
Full effect from the first dose	Full effect after repeated doses

### Currently Available P-CABs

Vonoprazan (TAK-438), first introduced in Japan and later in other Asian Countries, has recently been approved by US FDA and is on the market in North America and Central/South American Countries. Being in clinical use for more than 9 years, considerable clinical data have been accumulated and are detailed in several extensive reviews [15, 20, 26–32].

**Fig. 1:**
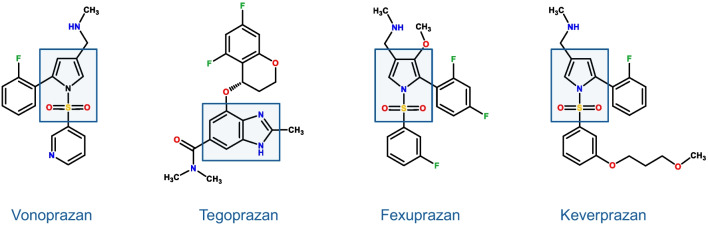
P-CABs currently available for clinical practice. Note that vonoprazan, fexuprazan and keverprazan share the sulphonylpyrrole structure while tegoprazan is a benzymidazole derivative. In addition, vonoprazan is given as fumarate whereas keverprazan is a hydrochloride

Tegoprazan (formerly RQ-00000004 or CJ-12420) was first introduced in 2019 into the Korean market and then brought to other Asian and Central/South American Countries [33, 34]. Fexuprazan (DPW14012) was approved in Korea in 2021 [35] and keverprazan (H008) received its first approval in China in 2023 [36]. While vonoprazan, keverprazan and fexuprazan are sulphonylpyrrole derivatives, tegoprazan holds the benzimidazole structure (Fig. 1).

### Vonoprazan: Clinical Pharmacology

PK and PD studies were performed in Japanese and Caucasian healthy male volunteers [37] and showed that vonoprazan displays almost linear pharmacokinetics and a dose-dependent inhibition of acid secretion. Both the 24-h and nocturnal pH > 4 holding times were linearly correlated with AUC [8]. The increase in pH was reflected by an increase in serum gastrin and pepsinogen I concentrations. These pharmacological effects persisted with repeated administration and, after 7 days of treatment, the mean 24-h intragastric pH > 4 holding time with vonozapran 40 mg was 100% in Japanese subjects and 93.2% in UK volunteers; mean nocturnal times spent at pH > 4 were 100% and 90.4%, respectively [38]. In *H. pylori*-negative healthy volunteers, the increase of intragastric pH with vonozapran (20 mg) was higher and faster compared to lansoprazole (30 mg) and similar to famotidine (20 mg) [39]. Vonoprazan was well tolerated at all doses tested, with no changes in serum transaminase levels.

**Fig. 2:**
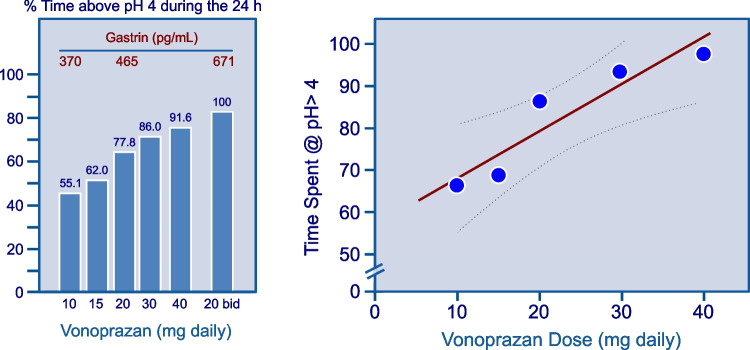
Antisecretory activity of vonoprazan: dose-related effect, as found in a a systematic review including 6 study arms and 864 patients (from Tansel & Graham [40])

The antisecretory effect of vonoprazan was quantified in a comprehensive systematic review including 6 study arms and 864 patients (Fig. 2). The analysis showed a linear relationship between drug dose and antisecretory activity [40] thus predicting a dose-dependent therapeutic efficacy in GERD [41].

Unlike esomeprazole, the antisecretory activity of vonoprazan was independent of CYP2C19 genotype [42]. In CYP2C19 extensive metabolizers, vonoprazan (20 mg) induced a more rapid and sustained acid inhibitory effect than esomeprazole (20 mg) or rabeprazole (10 mg), and showed virtually no episodes of nocturnal acid breakthrough (NAB) [43]. Therefore, vonoprazan (as well as other members of the P-CAB class) appears to be independent of CYP2C19 polymorphism, providing a similar acid suppression in both Asians and Caucasians. As a consequence, P-CABs overcome the PPI genotype-dependency in both PK and PD (especially for GERD and *H. pylori* eradication) [4] and allow for a more consistent effect in different patient populations [8].

By using a large data set of available PK studies in healthy volunteers and patients with GERD from Asia and Europe, we developed a population PK model, which was used to evaluate the impact of different covariates, (including race and disease status) on vonoprazan exposure [44]. PK parameters were similar in Asian and non-Asian populations. Variations in weight, age, and race were not predicted to have a clinically relevant impact on vonoprazan exposure or safety and require no changes in vonoprazan dosing. The limited impact of race on exposure suggests that the large body of data on efficacy and safety data for vonoprazan in Asian populations are translatable to non-Asian patients.

A recent crossover study [45] evaluated specifically the food effect on vonoprazan PK, by giving a single 20-mg dose of vonoprazan either following an overnight fast or 30 min after a high-fat breakfast. All the calculated PK parameters in fed and fasted conditions were not significantly different, indicating that vonoprazan can be administered without regard to food intake.

To evaluate the drug-to-drug interaction (DDI) potential of vonoprazan, both in vitro (rat liver microsomes) and in in vivo studies were performed [46]. The results indicated that vonoprazan can inhibit CYP3A4, CYP2C9, CYP2D6, and CYP2B6, suggesting that the co-administration of vonoprazan with cytochrome P450 substrates should be performed cautiously in any clinical setting.

A tiered approach was applied to understand the CYP3A victim and perpetrator DDI potential for vonoprazan [47]. A clinical study was conducted to evaluate the impact of vonoprazan on the exposure of oral midazolam, an index substrate for CYP3A. In addition, a physiologically-based pharmacokinetic (PBPK) model for vonoprazan was developed using all the available in vitro data and in vivo clinical data. The clinical midazolam DDI study indicated weak inhibition of CYP3A, with a less than twofold increase in midazolam exposure. PBPK simulations projected a 50% to 80% reduction in vonoprazan exposure when administered concomitantly with moderate or strong CYP3A inducers. On the contrary, poziotinib (a tyrosine kinase inhibitor employed in HER2 exon 20 mutant Non-Small Cell Lung Cancer, NSCLC), mainly metabolized by CYP3A4, was found to significantly inhibit the metabolism of vonoprazan [48]. Their co-administration should therefore be considered with caution.

Despite weak in vitro activity against CYP2C19, vonoprazan was found to attenuate the antiplatelet function of clopidogrel and prasugel [49]. To better understand the mechanism of this interaction, the effects of vonoprazan in comparison with esomeprazole (well known for its CYP2C19 Inhibitory activity [50]) were evaluated on the pharmacokinetics of a specific CYP2C19 substrate, proguanil [51]. Co-administration of both drugs resulted in increase and decrease in AUC of proguanil and its metabolite (cycloguanil), respectively. Therefore, like esomeprazole, vonoprazan potentially inhibits CYP2C19 at therapeutic doses, suggesting caution in the co-administration of these drugs with CYP2C19 substrates. As a matter of fact, in renal transplant patients vonoprazan co-administration increases tacrolimus concentrations [52]. Thus, frequent monitoring of blood tacrolimus concentration is required when vonoprazan is introduced as an antisecretory compound in the early phase of post-transplantation.

Due to their potent and long-lasting antisecretory activity, covering day and night, P-CABs soon became a component of the currently adopted *H. pylori* eradication regimens, administered in addition to antimicrobials. It is therefore of importance to know its PK and/or PD interactions with any other drug also used in the treatment of *H. pylori* infection. Compared with the P-CAB alone, triple therapy with vonoprazan-amoxicillin-clarithromycin increased the AUC_0-12h_ and C_max_ of plasma vonoprazan free base (by 1.8-fold), and increased the AUC_0-12h_ and C_max_ of plasma clarithromycin (by 1.5 and 1.6-fold, respectively). In contrast, triple therapy with vonoprazan-amoxicillin-metronidazole had no influence on the pharmacokinetics of vonoprazan or metronidazole. The pharmacokinetics of amoxicillin was not influenced by any vonoprazan-based triple therapy [53].

Along the same lines, since acid suppression is an effective gastroprotection in low-dose aspirin (LDA) and NSAID users, understanding of their mutual interaction(s) is of pivotal importance, especially in the elderly. A phase 2, open-label, study [54] evaluated drug-drug interactions between vonoprazan 40 mg and LDA (100 mg) or different NSAIDs [loxoprofen sodium (60 mg), diclofenac sodium (25 mg), or meloxicam (10 mg)] and viceversa. There were few differences in the PK of vonoprazan when administered with LDA or NSAIDs, and few differences in the pharmacokinetics of LDA or NSAIDs when administered with vonoprazan. These differences were small and not clinically relevant. Inhibition of arachidonic acid-induced platelet aggregation by LDA was not influenced by vonoprazan. These results justify gastroprotection with vonoprazan.

### Tegoprazan: Clinical Pharmacology

**Tegoprazan** (formerly RQ-00000004 or CJ-12420) is a benzimidazole derivative with potent and reversible inhibition of K^+^/H^+^-ATPAse, endowed with a strong and long-lasting antisecretory activity, which proved to be effective in experimental models of reflux disease and peptic ulcer [55, 56]. The first human study [57] showed that single oral administration of tegoprazan provides rapid elevation of intragastric pH to > 6 under fasted condition in healthy subjects. A subsequent dose-ranging study [58] demonstrated a linear PK profile after single and multiple oral dose administrations and a rapid and dose-dependent acid suppression. Its bioavailability was estimated to be 86–100%. The compound is mostly eliminated through the feces, with renal excretion limited to 3–6% [59]. When single doses of tegoprazan and revaprazan were compared, antisecretory the antisecretory activity of the former was significantly better, with a pH ≥ 4 holding time of 54.5 *versus* 25.1% achieved by the latter drug. However, the safety parameters (including those concerning liver function) were similar [60].

To improve patient compliance, an orally disintegrating tablet (ODT) of tegoprazan was recently made available. The PK profiles of both conventional tablet and ODT (with or without water) were reported to be equivalent [61]. In addition, a delayed release formulation of tegoprazan was also developed and the PK and PD of various combinations of immediate-release (IR) and delayed-release (DR) formulations carefully studied. The combination of the IR and DR formulation (1:1 ratio) was found to induce stronger gastric acid suppression throughout the day and at night, compared to the conventional IR formulation [62]. When approved, these new formulations will offer clinicians more therapeutic choices.

Two studies [63, 64] evaluated the effect of food on PK and PD of tegoprazan (single oral doses of 50 and 200 mg) and both showed that absorption was delayed under fed condition compared with that of the fasting condition. However, no significant differences were observed in the AUC and 24-h gastric acid suppression, indicating that tegoprazan could be administered regardless of the timing of food consumption and that it displays a meal-independent antisecretory effect. By incorporating in vitro metabolism and absorption profiles together with clinical data, a PK/PD model to predict food effect was developed [65]. Besides fitting the observed post-prandial PK profiles, this model provides a basis for evaluating changes in intragastric pH following tegoprazan administration.

Considering tegoprazan-based triple eradication therapy, a study evaluated the PK and PD of this P-CAB, when co-administered with amoxicillin/clarithromycin in healthy subjects [66]. PK analysis revealed a 2.1-times increase in AUC for both tegoprazan and its M1 active metabolite and the AUC of 14-OH clarithromycin, the antibiotic active metabolite increased 1.8 times while amoxicillin PK was not changed. On days 1 and 7 of treatment, tegoprazan-based regimens (both 50 and 100 mg therapies) maintained pH above 6 for more than 88% of the 24-h period, which was significantly longer when compared with pantoprazole-based therapy [66]. The reciprocal increase in plasma drug concentrations when tegoprazan and clarithromycin are administered in combination was later confirmed and better characterized by another PK study [67].

The DDIs during tegoprazan-based bismuth quadruple therapy can of course be more complex (due to the number of drugs and pills) and have been evaluated in a randomized, multiple-dose, crossover study [68]. The results showed that, while tegoprazan and tetracycline AUC were decreased, the bismuth absorption was increased during combination therapy. The PK of metronidazole was unchanged.

A randomized, multiple-dose, 3-way crossover study [69] evaluated drug-drug interactions between tegoprazan (50 mg once daily) and different NSAIDs [naproxen (500 mg b.i.d.), aceclofenac (100 mg b.i.d) or celecoxib (200 mg b.i.d.)] and viceversa. Both the C_max_ and the AUC of naproxen and celecoxib did not change significantly, while the C_max_ of aceclofenac increased by some 30%. However, the increase in AUC was very small (albeit significant) with no clinical relevance. On the other hand, NSAIDs did not change tegoprazan PK. These results confirm that gastroprotection with this P-CABs is feasible.

### Fexuprazan: Clinical Pharmacology

**Fexuprazan** (formerly DWP14012) is a sulpholylpyrrole derivative with potent and reversible inhibition of K^+^/H^+^-ATPAse, endowed with a more effective, stronger and long-lasting antisecretory activity compared to lansoprazole in several animal models [70] and displaying esophageal and gastric mucosal protection in experimentally-induced reflux disease and peptic ulcer [71]. The first human study was a dose-ranging randomized clinical trial (RCT), performed in healthy male, *H. pylori-*negative subjects [72]. The PK of fexuprazan after both single and multiple oral doses (20–160 mg) was linear and was not influenced by a high-fat meal. The drug achieved dose-dependent, rapid and sustained suppression of gastric acid secretion throughout the 24 h after single and multiple oral administrations, with the 40 mg and 80 mg doses showing no NAB [72]. Single- and multiple-dose administrations of fexuprazan were generally well tolerated by the subjects. The potential hepatotoxicity of fexuprazan, evaluated with serum levels of liver enzymes, total bilirubin and liver-specific microRNA-122 (miR122) [73], was not higher than that of placebo after multiple oral administrations. No clinically significant abnormalities in liver enzyme and total bilirubin levels were found. In a subsequent study, the PK and PD of fexuprazan were compared among Korean, Caucasian and Japanese healthy subjects [74]. The systemic drug exposure was similar between the three ethnicities after the 40 mg (approved) dose but slightly lower in Caucasian and Japanese subjects after the 80 mg dose. Gastric acid suppression showed a clear exposure–response relationship in all the three ethnicities.

Although the apparent PK of fexuprazan can be described by a simpler, physiologically-based pharmacokinetic (PBPK) models were developed [75, 76], which proved to be capable of effectively simulating the observed data and, by integrating the effects of perpetrator drugs, can be used to predict the impact of drug-to-drug interactions.

With the aim of employing fexuzapran for gastroprotection, a randomized, open-label study evaluated the PK and PD interactions between aspirin (500 mg) and fexuprazan (80 mg) in healthy Koreans [77]. Neither aspirin-induced inhibition of platelet aggregation nor systemic exposure to aspirin were significantly affected by fexuprazan coadministration. The systemic exposure of fexuprazan was decreased up to 20% by aspirin co-administration, but this was not considered clinically relevant.

### Keverprazan Clinical Pharmacology

Keverprazan hydrochloride represents the first P-CAB developed in China. Despite preclinical pharmacology data have not yet been published, PK and PD data are available and phase III clinical trials on healing of reflux esophagitis and duodenal ulcer have been performed. The results of these clinical studies allowed the Regulatory Authority to grant it the approval for these indications [36].

The PK study on keverprazan [78] showed quick absorption (with T_max_ ranging from 1.25 to3.0 h) and a terminal half-life at steady state of 6.23 and 7.01 h for the 20 and 40 mg dose, respectively. There was no apparent accumulation of keverprazan and the major metabolite after 7-day administration. The drug induced a rapid increase of intragastric pH (reaching pH 4 in about 2 h), which was stable over the 24 h, with a pH ≥ 5 holding time of 97.4% and 100.0% at steady state for the 20 mg and 40 mg dose, respectively. Under the same experimental conditions, the same parameter for vonorazan (20 mg dose) was 99.7%.

Compared with fasting, the mean Cmax and AUC of keverprazan in the fed conditions was increased by 27% and 35%, respectively, showing that drug exposure is increased when it is taken after a high-fat breakfast meal (800–1000 cal, with approximately 50% of total caloric content from fat). There was no obvious food effect on T_max_ or half-life [79].

### P-CAB Head-to-Head Comparison

As with PPIs, for which only limited head-to-head comparative data exist despite four decades of clinical use, the challenge ahead for newer drugs of this very effective pharmacologic class will be to point out the differential characteristics of each individual drug.

**Fig. 3:**
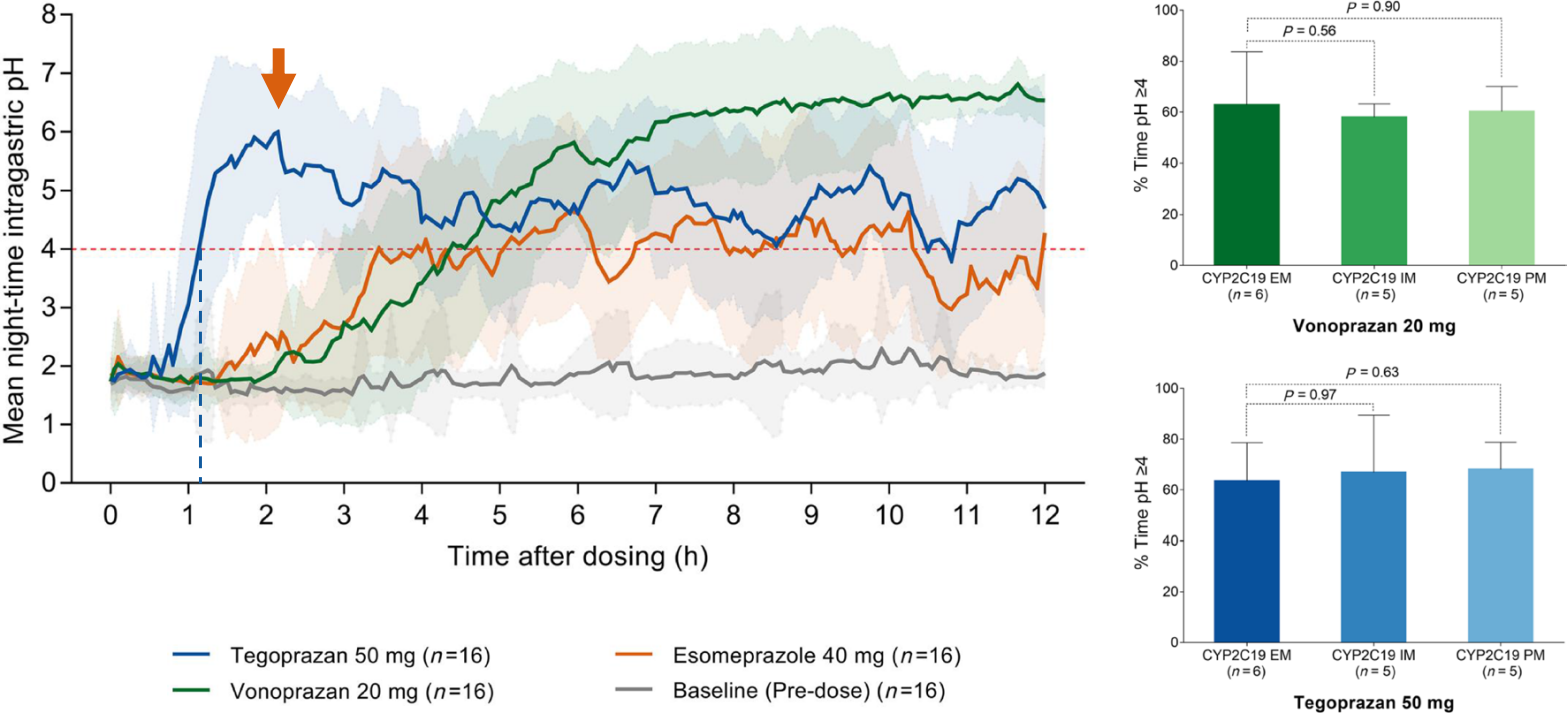
Effect of evening doses of tegoprazan, vonoprazan or esomeprazole on nocturnal acidity in healthy volunteers (*modified* from Yang et al*.*, [80])

In a recent study [80] the effect of tegoprazan, vonoprazan or esomeprazole on nighttime acid suppression was compared in healthy volunteers, (6 CYP2C19 extensive metabolizers, 5 intermediate metabolizers, 5 poor metabolizers). When administered at bedtime, tegoprazan induced a more rapid inhibition of nocturnal acid secretion compared to vonoprazan or esomperazole. However, over time, vonoprazan produced higher and more sustained elevations of intragastric pH. Both P-CABs showed no NAB (Fig. 3). Furthermore, tegoprazan and vonoprazan effects were independent from CYP2C19 polymorphism [80]. Similar results were obtained when tegoprazan (50, 100 and 200 mg) was compared with dexlansoprazole [81]. A recent network meta-analysis [82], including 55 RCTs and 2015 subjects, compared the efficacy of different antisecretory regimens in preventing NAB. Overall, amongst them (H_2_RAs, first- and second-generation PPIs, H_2_RAs at bedtime, novel PPIs and P-CABs) vonoprazan and tegoprazan showed the highest cumulative rank probability (> 90%) of night-time acid suppression.

As for individual members of other drug classes, together with PD, DDI differences are starting to emerge. When atorvastatin was co-administered with vonoprazan, the systemic exposures of atorvastatin and atorvastatin lactone significantly increased while this was not the case for tegoprazan [83]. This difference in behavior has been attributed to the luminal concentrations of vonoprazan (compared to tegopranzan) which were increased enough to inhibit intestinal CYP3A4 and increase the systemic exposure of the orally administered statin.

Since, in both healthy volunteers and patients with GERD, serum gastrin and pepsinogen I levels mirror the antisecretory effect of PPIs and P-CABs [84, 85], hypergastrinemia associated with long-term therapy might cause a concern. This issue was extensively addressed at the Hanbury Manor Workshop in 1995 [86] and there has been no convincing evidence of neoplastic change in the subsequent 30 years of follow up [87]. In most studies of hypergastrinemia associated with the use of antisecretory drugs, gastrin levels do not continue to increase and promptly return to normal after discontinuation of therapy [88]. Despite almost similar antisecretory effect, P-CABs appear to induce a different degree of hypergastrinemia, which is higher for vonoprazan compared to tegoprazan as well as to fexuprazan and zestaprazan [89]. The underlying reasons are not clear at the present time and further data are needed.

### Current Clinical Indications of P-CABs

Although vonoprazan was approved in Japan for a wide range of acid-related diseases, including gastric and duodenal ulcer, reflux esophagitis, prevention of LDA and NSAID-associated ulcer and, in combination with antimicrobials, *Helicobacter pylori* infection and related conditions [20], the most widely accepted clinical use in North and South/Central America are erosive and non-erosive reflux disease as well as *H. pylori* eradication.

Since all the (approved and potential) vonoprazan indications have been presented in a previous review [15], only GERD and *H. pylori* infection, will be discussed below.

## Vonoprazan Efficacy in GERD

### Erosive Reflux Disease

As predicted by a large meta-analysis evaluating the intra-gastric pH data of the currently used antisecretory regimens [41, 90], the healing rate of reflux esophagitis after 8-week therapy with vonoprazan was very high (almost 100% in Asian trials). The large amount of clinical data accumulated have been the subject of some systematic reviews and meta-analyses, providing evidence-based results.

While another meta-analysis is still ongoing [91], a systematic review and meta-analysis [92] including 6 eligible RCTs, comparing the efficacy and safety of vonoprazan with PPIs for GERD, has been published. Results show that vonoprazan is non-inferior to PPIs as therapy for patients with GERD (RR: 1.06 – 95% C.I. 0.99–1.13). However, subgroup analysis indicates that vonoprazan is more effective than PPIs for patients with severe erosive esophagitis (RR: 1.14 – 95% C.I. 1.06–1.22). The safety outcomes for vonoprazan are similar to those for PPIs (RR: 1.08 – 95% C.I. 0.96–1.22). In addition to *pairwise* comparisons, three *network* meta-analyses are available [93–95]. The first [93] shows that GERD-healing with vonoprazan is higher than with rabeprazole (20 mg) but not higher than other PPIs. However, subgroup analysis indicates that vonoprazan is more effective than most PPIs for patients with *severe* erosive esophagitis. This was confirmed by the second, very recent meta-analysis [94], specifically devoted to grade C and D (according to the Los Angeles classification) esophagitis. Based on the failure to achieve mucosal healing, 20 mg of vonoprazan q.d. ranked first among antisecretory drugs in initial and maintained healing of severe mucosal lesions. The superiority of vonoprazan over some PPIs in the maintenance of healing was confirmed also by the third network meta-analysis [95], which however suggested future direct comparisons to confirm this finding.

**Fig. 4:**
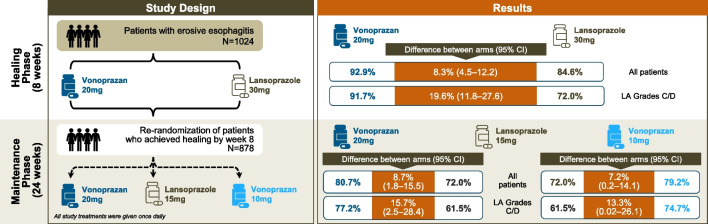
Vonoprazan *versus* lansoprazole for healing and maintenance of healing of erosive esophagitis: experimental design and results from the US trial [from Laine et al*.* [97])

Two large non-inferiority RCTs [96, 97], not included in the above systematic reviews, have recently been published. One in Asia (predominantly mainland China, Malaysia, South Korea and Taiwan) and one in USA. In the Asian trial [96] vonoprazan 20 mg was shown to be effective and non-inferior to lansoprazole 30 mg in terms of endoscopic reflux esophagitis healing rate at 8 weeks, with slightly higher healing rates of vonoprazan at 2 and 4 weeks. The US trial [97], the first comparing a P-CAB and a PPI in a *H. pylori*-negative Western population, confirmed that vonoprazan was non-inferior to lansoprazole for healing and maintenance of healing of erosive esophagitis. Furthermore, it showed that this P-CAB achieved higher rates of healing and maintenance of healing than the PPI, with the differences seen primarily in those with more (C and D) severe esophagitis (Fig. 4). In both trials, there was no statistically significant difference in symptom relief between the treatment arms. However, in some studies [98] significantly more patients attained complete nocturnal heartburn relief with vonoprazan than with lansoprazole.

By combining the data from the above trial with previous Japanese and European phase 1 studies, we generated a PK/PD model to link vonoprazan exposure to pH holding time and to evaluate the dose-exposure–pH holding time ratios (HTR) relationship [99]. The model, validated by goodness-of-fit plots between predicted and observed pH values, allowed simulations to predict pH HTRs with vonoprazan 20 mg once and twice daily at day 7 and showed that these doses should maintain intragastric pH > 4 for 89.7% and 98.1% of the time, respectively. These data support the use of the above regimens in clinical practice either for GERD or *H. pylori* infection (*see below*).

Esophageal mucosal injury is often accompanied by symptoms such as heartburn, regurgitation, sore throat, and swallowing difficulties [100]. Besides healing erosive esophagitis, reflux symptoms should also be managed since they have a detrimental impact on health-related quality of life (HRQoL). Therefore, the appropriate drug able to achieve as quickly as possible complete symptom relief should be selected. Since vonoprozan displays a fast onset of antisecretory activity, it should be expected to provide a similarly fast symptomatic effect. Unfortunately, symptom resolution has only seldom specifically evaluated in clinical trials. A double-blind, placebo controlled, RCT [98] showed that heartburn was relieved sooner with vonoprazan than with lansoprazole (p < 0.05). Heartburn was completely relieved in 31.3% and 12.5% of patients on day 1 with vonoprazan and lansoprazole, respectively. Furthermore, significantly more patients achieved complete nocturnal heartburn relief with vonoprazan than lansoprazole (p < 0.01). Due to lack of head-to-head comparisons (with the exception of this trial), a network meta-analysis [101] was needed to provide an evidence-based evaluation of vonoprazan speed of action. By including 10 RCTs, specifically assessing symptom relief in patients with reflux esophagitis after acid suppression, the analysis found that—for heartburn resolution rate on day 1—vonoprazan (20 mg once daily) was superior to placebo (median odds ratio = 16.75, 95% credible intervals: 2.16–207.80). For heartburn resolution rate on day 7, vonoprazan was superior to placebo and other comparators except rabeprazole (20 mg once daily).

Some small studies have shown that vonoprazan is also effective in patients with PPI-resistant esophagitis. A recent systematic review with meta-analysis [102], which included both observational and clinical studies (N = 12), found the drug effective both in treatment and maintenance of this challenging clinical condition. Healing rates of PPI-resistant erosive esophagitis with vonoprazan 20 mg were 91.7% (95% CI: 86.8–94.8%) and 88.5% (95% CI: 69.7–96.2%) at weeks 4 and 8, respectively. Healing was accompanied by symptom relief in a large proportion (some 75% at week 4) of patients. The 10 mg dose was also effective in maintenance of healing in 86.0% (95% CI 72.1–94.7%) of patients at week 24, and 93.8% (95% CI 69.8–99.8%) at week 48.

### Non-erosive Reflux Disease

Several Asian studies (*for review see* [15]) have shown that, besides in erosive esophagitis, vonoprazan (given daily or on demand) is effective in non-erosive disease (NERD) as well. A recent US trial [103] evaluated the efficacy and safety of different doses of vonoprazan versus placebo for the on-demand treatment of patients with NERD efficacy of different doses (10, 20 and 40 mg) of vonoprazan *versus* placebo for the on-demand treatment (drug taken in response to heartburn episode) of patients with NERD. All the investigated doses were safe and significantly better than placebo in providing rapid and sustained relief from heartburn episodes, with the 40 mg dose, providing limited extra benefit over the lower doses. Although the primary endpoint was assessed at 3 h, greater improvement in heartburn was already observed within the first hour in the vonoprazan group as compared to the placebo group and occurred within 30 min [104]. Thanks to its favorable PK and PD, preventive consumption 1–2 h before) of vonoprazan may be desired and practiced by some patients who anticipate GERD-related symptoms after a large meal.

It is important to appreciate that P-CAB-resistant NERD has also been reported and ascribed to weakly acidic reflux or much more likely to be functional heartburn [105]. This *apparent* resistance, however, could be dose-dependent. A retrospective, small study [106] evaluated NERD patients with symptoms resistant to double-dose PPIs, who were switched to vonoprazan (20 mg daily). pH-impedance recording revealed fewer reflux events at pH < 5 in patients with symptom improvement compared to those without. In these patients, the proportion of reflux at pH < 4 decreased but that of reflux at pH 4–5 increased while that of reflux at pH < 5 did not change [106]. Despite the limitations of the study, the results suggest that the lack of symptom improvement may be related to inadequate acid suppression that could be addressed by a higher vonoprazan dose.

## Tegoprazan Efficacy in GERD

### Erosive Reflux Disease

The first Korean trial [107] demonstrated that tegoprazan, administered at 50 or 100 mg once daily, was non‐inferior to esomeprazole 40 mg in achieving healing rate of erosive esophagitis at both week 4 (90.3% *versus* 88.5%) and week 8 (99.1% *versus* 99.1%). Both doses of tegoprazan were highly effective albeit the 100 mg dose provided no additional clinical benefit over 50 mg. The number of patients with C and D esophagitis was too small to allow a subgoup analysis with proper interpretation of results in severe esophageal injury.

Tegoprazan (25 mg daily) was also non inferior to lansoprazole (15 mg daily) for maintaining remission of healed mild esophagitis at 12 and 24 weeks, an efficacy that remained consistent in CYP2C19 extensive metabolizers [108]. The endoscopic remission rate after 24 weeks was 90.6% with tegoprazan and 89.5% with lansoprazole. Tegoprazan was not inferior to lansoprazole also for maintaining endoscopic remission also at 12 weeks.

### Non Erosive Disease

Like vonoprazan, tegoprazan was also found to be effective in patients with NERD. Two doses of the P-CAB (50 and 100 mg) were tested and both were found more effective than placebo, with 42.5%, 48.5% and 24.2% of patients in each arm respectively, showing complete resolution of major symptoms (heartburn and regurgitation) at week 4 [109]. The tegoprazan response rate was similar to that observed with PPIs, which are known to be less effective in endoscopy-negative reflux disease due to the complex pathophysiology and heterogeneous population of patients [110].

### Fexuprazan Efficacy in GERD

This P-CAB was investigated in a phase 3, multicenter, randomized, double-blind trial. Two hundred and sixty adult patients with endoscopically confirmed erosive esophagitis (LA Grades A to D) were randomized to receive fexuprazan (40 mg once daily) or esomeprazole (40 mg once daily) [111]. The primary outcome measure was the cumulative proportion of patients with healed mucosal breaks, confirmed by endoscopy, at week 8. Healing rate at week 4, symptoms and quality of life were also assessed. Fexuprazan was non-inferior to esomeprazole, with identical (i.e., 99.1%) cumulative healing rates at 8 weeks and similar rates (90.3% and 88.5%, respectively) at 4 weeks. However, fexuprazan showed better symptom relief in patients with moderate to severe heartburn, an effect persisting also during night time. A similar benefit was evident also for cough. The drug was well tolerated, with an incidence of adverse events comparable between treatment groups [111]. Another non-inferiority study with a similar experimental design was performed in China [112]. The healing rates of fexuprazan and esomeprazole groups at 8 weeks were 88.5% and 89.0%, respectively. No significant difference was found between groups in esophagitis healing rates at 4 weeks, as it was in symptom responses and changes of GERD-HRQL. Two additional studies with fexuprazan were presented at the 2023 Digestive Disease Week meeting. The first [113] confirmed that this P-CAB provides the esophagitis healing rate and symptom relief of esomeprazole. In the second study [114], the effect of two different dosing times (before-meal and after meal) of fexuprazan (40 mg once daily) in patients with erosive esophagitis were compared. Healing rates were similar between the two arms at both 2 weeks (95.8% *versus* 97.1%) and 4 weeks (98.8% *versus* 100%).

### Keverprazan for GERD

**A p**hase III RCT [115], performed in China, compared keverprazan (20 mg once daily) to lansoprazole (30 mg once) for healing erosive esophagitis. This study demonstrated the non-inferior efficacy and safety of keverprazan to lansoprazole. Esophagitis healing rates at 4 and 8 weeks were comparable between the two arms, with healing rates of 95.8% and 89.9%, respectively. However, per protocol analysis showed that the keverprazan group even had a significant higher healing rate at 8 weeks.

### P-CABs for GERD: Conclusions

P-CABs clearly overcome many of the drawbacks and limitations of the DR-PPIs. In acid-related disorders, mucosal healing is directly related to the degree and duration of acid suppression and the length of treatment [13, 41]. Considering the difficulties encountered in achieving effective symptomatic control, particularly at night, using currently available DR-PPIs once daily, this new class of drugs achieves rapid, potent and prolonged acid suppression and offers the chance of addressing some of the unmet clinical needs in GERD [6, 10, 11, 13], such as the need for fast and assured healing of severe reflux esophagitis and achieving rapid heartburn relief.

**Fig. 5:**
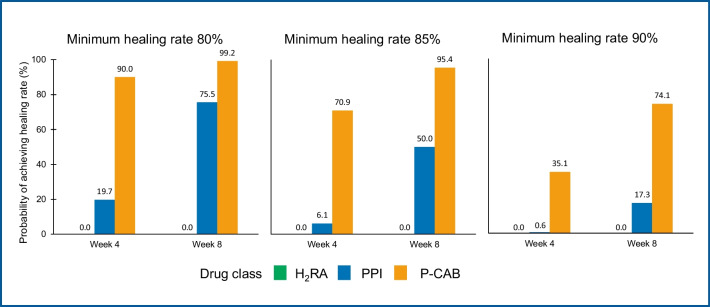
Probability to achieve a target healing rate by the different classes of antisecretory drugs (from Howden et al*.* [116])

We have recently developed a mathematical (non-linear, mixed-effects) model to examine the relationship between pH holding HTRs and erosive esophagitis healing rates with all the three classes of antisecretory drugs (H_2_RAs, PPIs and P-CABs) [116]. Data from 82 papers, reporting mean pH > 4 HTRs at steady state, and 104 clinical trials with esophagitis healing rates, were included in the analysis. The final model well described the available data and the correlation between observed and predicted healing rates was excellent. By using this model, we were able to calculate the probability to achieve a target healing rate by the different classes of antisecretory drugs. The results show clearly that – whatever the selected target – P-CABs appear the most effective drugs for healing reflux esophagitis (Fig. 5).

On the basis of their superior efficacy, P-CAB treatment is now suggested for healing of mucosal lesions by the most recent Asian guidelines on GERD (the Seoul Consensus [117], the Chinese Expert Consensus [118], the Japanese Guidelines [119]) and by the very recent Mexican Clinical Practice Recommendations [120] and will surely be included in the future recommendations from the Western GI Societies.

### P-CABs for the Treatment of *H. pylori* Infection

Currently PPIs are an essential component of any *H. pylori* eradication regimen [121]. To be most effective, full dose PPIs should be given twice daily, concomitantly with antimicrobials since the mean cure rates are greater in patients who use high-dose PPI, compared with the standard-dose regimen [4]. The importance of the *degree* of acid suppression on the eradication efficacy became apparent when attempting to validate a dual (namely omeprazole-amoxicillin combination) therapy. Indeed, a linear relationship between omeprazole dose (20–120 mg daily) and eradication rate was clearly evident: the greater acid suppression, the higher eradication rate [122].

Prolonged acid suppression especially during the night, is crucial for *H. pylori* eradication [123, 124]. Moreover, it was clear that the eradication rate was higher in those without nocturnal acid breakthrough (NAB) than in NAB-positive patients [123]. As a consequence, antisecretory drugs, which offer superior control of both day and night-time acidity, should represent a better choice for *H. pylori* eradication regimens.

### Vonoprazan-based Eradication Regimens

A large amount of data on the use of vonoprazan as a component of different eradication regimens have now been accumulated from studies in Japan and other Asian Countries. Only recently has the drug been investigated in US and European patients.

In contrast to PPIs [4], which display a direct antimicrobial activity against *H. pylori*, vonoprazan (as well as tegoprazan) do not inhibit the growth of the microorganism [125]. However, the minimal inhibitory concentrations (MICs) of clarithromycin, fluoroquinolone, metronidazole, and amoxicillin against resistant *H. pylori* isolates improved after tegoprazan administration. The proportion of strains affected varied from 35 to 56%, depending on the given antimicrobial.

The better efficacy of vonoprazan-based, over PPI-based triple (and, more recently, quadruple) regimens – *as first line therapy*—has been emphasized by several meta-analyses [126–130]. In addition, a systematic review of *second line treatments* [131] showed that vonoprazan-based regimens still provide significantly higher eradication rates compared to PPI-based regimens. Moreover, an additional meta-analysis [132] found that vonoprazan is superior to conventional PPIs only for eradication of clarithromycin-resistant *H. pylori* strains while vonoprazan-based and conventional PPI-based therapies are similarly effective in patients harboring clarithromycin-susceptible *H. pylori* strains. Finally, an interesting, albeit retrospective, study [133] found that vonoprazan-based triple therapy is effective as susceptibility-guided triple therapy for *H. pylori* eradication.

After the original report of Miehlke et al*.* [122], several investigators studied both standard- and high-dose PPI combinations with amoxicillin in the hope of finding an effective and simple eradication regimen. While the standard dose PPI-amoxicillin dual therapy gave disappointing eradication rates (*for review see* [134]), stronger and long-lasting acid suppression appeared to be successful. Nine meta-analyses [135–143] collected all the studies with *high-dose* PPI-amoxicillin combinations, which showed that this dual therapy is as effective as triple or bismuth-based quadruple therapy, either in first-line or rescue treatment. In addition, compliance with dual therapy was in some analyses better and the adverse event rate always lower [135–137].

The antisecretory effect of vonoprazan is long-lasting (covering both daytime and nighttime). A twice daily dose could therefore be sufficient to synergize with amoxicillin, thus further enhancing compliance with respect to the high-dose PPI regimens. As attested by 6 different meta-analyses [144–149], the eradication rate of vonoprazan-amoxicillin dual therapy was significantly higher than that of PPI-triple therapies. However, when this dual therapy was compared to bismuth-based quadruple therapy, two very recent meta-analyses provided different results According to the first [150], the cure rate of both regimens was similar while the second one [149] found a lower efficacy with dual therapy. Nevertheless, both studies pointed out that the rate of adverse effects of the dual therapies was significantly lower than the triple.

Together with pairwise meta-analyses, a large network meta-analysis, including 68 RCTs giving a total of 92 paired comparisons with 22.975 patients randomized to 8 first-line regimens, was performed by a group of European leading investigators [151]. The overall results showed that only vonoprazan triple therapy achieved cure rates of > 90%. Furthermore, the comparative effectiveness ranking showed that vonoprazan triple therapy gave the best results, whereas standard triple therapy was the least efficacious regimen. A more recent, network meta-analysis [152], including 101 *Chinese* trials involving 21.745 patients, found that the efficacy of vonoprazan-bismuth–containing quadruple therapy ranked first, followed by high-dose vonoprazan-amoxicillin dual therapy.

The first vonoprazan study for *H. pylori* eradication in the Western world was performed in Australia [153] by Borody’s Team, who evaluated eleven different antimicrobial combinations. These included different antibiotics (mainly amoxicillin, rifabutin and tetracycline) as well as levofloxacin, furazolidone, nitazoxanide They treated 153 patients, 31% of whom had previously failed eradication attempts with a PPI-based triple therapy. In patients treated for the first time, eradication was achieved in 100% of cases and in those, who had failed prior, non-vonoprazan-containing treatments, eradication was achieved in 91% of patients.

**Fig. 6:**
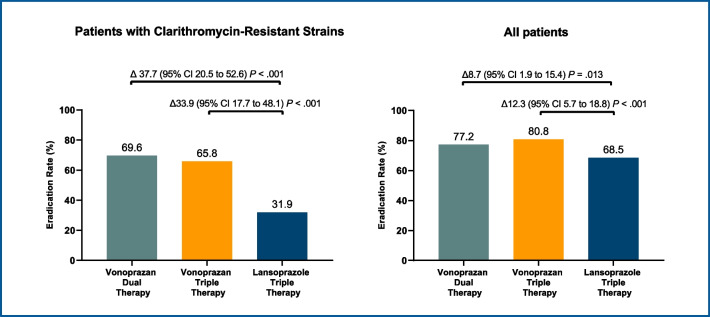
*H. pylori* eradication rates with vonoprazan-based therapies. Secondary outccomes from the US and European Trial (from Chey et al*.* [154])

A large, multicenter, RCT [154] assessed the efficacy of vonoprazan triple and dual therapy for *H. pylori* infection in the United States and Europe. A total of 1046 treatment-naïve, infected adults were randomized 1:1:1 to open-label vonoprazan dual therapy (20 mg vonoprazan twice daily; 1 g amoxicillin 3 times daily), or double-blind triple therapy twice a day (vonoprazan 20 mg or lansoprazole 30 mg; amoxicillin 1 g; clarithromycin 500 mg) for 14 days. Among patients from the United States and Europe, vonoprazan-based triple and dual regimens were non-inferior to lansoprazole-based triple therapy for eradication of *H. pylori* strains not resistant to clarithromycin and amoxicillin. In secondary analyses, vonoprazan triple and dual regimens achieved significantly higher eradication rates in the subgroup with clarithromycin-resistant strains and in the overall study population (Fig. 6). These findings were confirmed by the most recent meta-analysis, including 13 studies [155]: the overall efficacy of vonoprazan based therapy was superior to PPI-based therapy (RR: 1.09, 95% CI 1.03 to 1.05, p < 0.01). The same holds true in patients with clarithromycin-resistant strains (RR 1.64, 95% CI 1.21 to 2.23, p < 0.01).

Despite the cure rate of vonoprazan-amoxicillin combination was lower in Caucasian compared to Asian patients, dual therapy may become a simple, first-line regimen for the eradication of *H. pylori* infection. Before being widely adopted, this dual therapy should be optimized, by selecting the best dose, number of drug administrations and duration [29]. There are indeed sufficient data to prove that optimized vonoprazan-amoxicillin dual therapy can reliably achieve cure rates ≥ 95% [156].+

Antimicrobials have a detrimental effect on gut microbiota [157] and *H. pylori* eradication regimens make no exception [158]. In this connection, the dual amoxicillin therapy appears to have a minimal impact on the microbiota. Indeed, in contrast to vonoprazan-triple therapy, the alfa-diversity at 1 and 8 weeks did not change significantly compared with baseline [159].

In 2017 WHO listed *Helicobacter pylori* among 16 antibiotic-resistant bacteria that pose the greatest global threat to human health [160]. Given the alarmingly high *H. pylori* antibiotic resistance rates, antibiotic stewardship programmes need to be developed and implemented. In this regard, a move to dual therapies – by using only one antibiotic – will fit well to support this endeavor.

### Tegoprazan-based Eradication Regimes

On the basis of in vitro data [125] and of the vonoprazan experience (*see above*), tegoprazan (50 mg b.i.d.) was adopted as the antisecretory component of triple or quadruple therapies and compared with the PPI-based same regimens.

A large, RCT [161] evaluated tegoprazan-based classic 7-day triple therapy (with amoxicillin and clarithromycin) in comparison to the same lansoprazole-based combination. The *H. pylori* eradication rates were 62.7% and 60.6%, respectively. Subgroup analyses according to MICs or CYP2C19 did not show remarkable differences in eradication rate. A real-world study [162] reported overlapping results (eradication rate of 63.9%), which improved to 78.6% cure rate when extending the tegoprazan-triple therapy to 14 days. When an IR formulation of esomeprazole was used instead of lansoprazole, the eradication rate was similar to that found with tegoprazan-based regimen (78.6% *versus* 81.4%). These results were replicated in a study comparing tegoprazan- versus rabeprazole-based triple therapy (76.7% versus 75.4%, respectively) [163].

In an attempt to increase the efficacy of the above eradication regimes, bismuth subcitrate was added to both tegoprazan-based and lansoprazole-based 7-day triple therapy [164]. The corresponding eradication rates were 78.8% and 74.5%. A subsequent trial compared tegoprazan therapy, with and without addition of bismuth, showing that Bi-based therapy is significantly more effective (eradication rate: 82.9% *versus* 71.8%, p = 0.0029) [165].

Tegoprazan was also tested with the classic bismuth quadruple therapy (PPI + tetracycline + metronidazole + bismuth subcitrate) or concomitant therapy (PPI + amoxicillin + clarithromycin + metronidazole). When compared to a lansoprazole-based bismuth regimen, the tegoprazan-based therapy achieved a similar eradication rate (80% versus 77.4%) [166]. Ten-day tegoprazan-based concomitant therapy gave an eradication rate of 90.5% [167], which was not improved by extending the treatment at 14 days [168].

Despite tegoprazan-containing therapies meeting the criteria for non-inferiority to PPI-based therapies, they did not achieve the clinically acceptable cure rate of ≥ 95% or even conditionally acceptable threshold of 90–94% [169]. It should be appreciated, however, that all these studies have been performed in South Korea, where antimicrobial resistance and multidrug resistance are relatively high and still increasing [170, 171].

### P-CABs for the Treatment of *H. pylori* Infection: Conclusions

The distinctive PK and PD properties of P-CABs make them the antisecretory drugs of choice as the component of eradication regimens. In particular, they induce an increase of intragastric pH, which is greater and longer-lasting when compared with PPIs. This allows the microorganism to reach the growth phase, becoming more sensitive to antibiotics (such as amoxicillin and clarithromycin) and improves their intragastric stability and antibacterial efficacy [4].

Antimicrobial resistance is increasing worldwide and remains an important clinical challenge [172]. Vonoprazan use was already discussed in 2016 in the Toronto Consensus [173] and—based on the available data—the Maastricht VI/Florence consensus report [174] stated that “P-CAB-antimicrobial combination treatments are superior, or not inferior, to conventional PPI-based triple therapies for first-line and second-line treatment, and superior in patients with evidence of antimicrobial resistant infections” and suggested P-CAB use as component of dual, triple and quadruple therapies, where available.

### P-CABs in Clinical Development

**Linaprazan Glurate** (X842) is a pro-drug of linaprazan, developed in Europe by Cinclus Pharma AG. The active metabolite has a comprehensive data base from 25 Phase I studies, including more than 600 subjects, and 2 Phase II studies, including 2.973 patients. All these investigations showed linaprazan was well tolerated, with a fast onset of action and full effect from the first dose. However, linaprazan did not control 24 h intragastric pH, likely because of its short plasma half-life [175]. In contrast, linaprazan glurate has a longer half-life which provides effective 24 h pH control.

The first human study of X842 [176] evaluated the PK and PD after single and multiple ascending doses. Linaprazan rapidly appeared in plasma, with the C_max_ at ~ 2 h after oral administration. Plasma half-life was ≥ 10 h, following doses of 1 mg/kg or higher. Linaprazan AUC linearly correlated with the X842 dose, with a dose-dependent acid inhibition over the 24 h, and linear correlation between plasma concentrations of the active metabolite (i.e., linaprazan) and intragastric pH. At doses of 2 mg/kg, X842 achieved effective acid control over 24 h without evidence of NAB.

A phase 2, double-blind RCT [177] evaluated a dose-ranging (25–50-75 100 mg twice daily) healing effect of A/B and C/D erosive esophagitis in comparison with lansoprazole (30 mg once daily). The global healing rate for patients with A/B esophagitis was 83.8% and 81.0%, respectively. However, the healing rate for severe (C/D) esophagitis was fairly different, being 73.6% for linaprazan glurate and 37.5% for lansoprazole. The highest healing rate was seen with 75 mg dose that will likely be selected for phase III clinical trials. The drug was well tolerated with no dose-related increase in adverse effects, and the safety profile was comparable to that of lansoprazole [178].

The last P-CAB reaching clinical trials is **zastaprazan** (JP-1366, under development by Onconic Therapeutics in South Korea), a potent, competitive and highly selective inhibitor of H^+^/K^+^ ATPase, endowed with an effective and long-lasting antisecretory activity in several animal models and displaying esophageal and gastric mucosal protection in experimentally-induced reflux disease and peptic ulcer [179]. When compared, under the same experimental conditions, with vonoprazan, it appeared to be about 2.5 more potent. Furthermore, zastaprazan showed a faster onset of action.

The first human study was a dose-ranging RCT, performed in healthy male, *H. pylori-*negative Korean subjects [180]. The PK of zastaprazan after both single and multiple oral doses (20–160 mg) was linear and was not influenced by a high-fat meal. The drug achieved dose-dependent, rapid and sustained suppression of gastric acid secretion throughout the 24 h after single and multiple oral administrations, with the 20, 40 and 60 mg doses showing no NAB. Administration of zastaprazan after a high-fat meal decreased the peak plasma level while the overall systemic exposure of zastaprazan increased compared to those in the fasted state. The drug was well tolerated with no clinically significant changes in safety and tolerability assessments, with no appreciable changes in the liver function. The serum liver transaminases and fold change of miR-122 after single and multiple administrations of zastaprazan remained within the reference range and were not higher than that of the placebo [180].

## Conclusions and Future Perspectives

Effective control of intragastric acidity is crucial for the treatment of acid-related disorders including erosive esophagitis [41, 90, 116]. Although PPIs have been long considered the mainstay of treatment for erosive and non-erosive GERD, not all patients achieve healing of erosive esophagitis with a standard 8-week course and fewer actually achieve satisfactory symptom control. In addition, some patients will have relapse of esophageal lesions despite continuous once-daily maintenance treatment with a PPI. Since PPIs are usually dosed in the morning, many patients will show NAB that is often associated with nocturnal heartburn and poorer clinical outcomes [6, 10, 11].

Intragastric pH must also be effectively controlled for successful eradication of *H. pylori* infection [121, 181]. While a pH ≥ 7 is not pharmacologically achievable in the human stomach, prolonged duration of an intragastric pH > 6 is likely to be important for the eradication of the infection. A cut-off of an average intragastric pH of 6 across 24 h predicts successful eradication [124]. After the introduction of PPI-based triple therapies in the early ‘90s there has been a slow progress to optimizing eradication regimens with antisecretory drugs. Indeed, despite the advent of sequential, quadruple, concomitant and hybrid therapies, control of intragastric acidity remained suboptimal.

The distinctive pharmacological properties of P-CABs, described above, offered the chance of addressing many of the unmet needs in GERD and *H. pylori* infection [13, 16]. And indeed, this new class of antisecretory drugs has shown to be successful in these two clinical conditions that are now considered established indications [15].

Being a pH-dependent phenomenon [182], NSAID-gastropathy is effectively prevented by P-CABs [183–185], but their superiority over DR-PPIs has not been demonstrated in this clinical setting. However, based on the available evidence, also secondary prevention of NSAID-gastropathy can be considered an established indication (Table 3).

**Table 3:** Clinical indications (established, potential and under evaluation) of P-CABs

Indications	Superiority over PPIs
*Established*	
Severe (Los Angeles C & D) reflux esophagitis	Yes
Reflux (Los Angeles A & B) esophagitis	No
Eradication of the *H. pylori* infection	Yes
Secondary prevention of NSAID-gastropathy	No
Non erosive reflux disease	Possible
*Under Evaluation*	
Eosinophilic esophagitis	Likely No
Treatment of NSAID ulcer	Likely No
Idiopathic peptic ulcer	Likely Yes
Prevention of ESD delayed rebleeding	Likely Yes
*Potential*	
Treatment of upper GI (non-variceal) bleeding and prevention of rebleeding	Likely Yes

The relative efficacy of P-CABs is based on the current available evidence (meta-analyses, RCTs, observation studies and case reports), discussed in this review

Other uses (e.g., peptic ulcer, endoscopic mucosal dissection-induced ulcer, functional dyspepsia) of this class of drugs are being evaluated [15, 186, 187], but clinical data are not yet sufficient to allow a definitive answer on its efficacy and eventual superiority over our current standard of care (i.e., PPIs). The most important indication of upper GI (non-variceal) bleeding, where P-CABs are likely to outweigh the benefits of DR-PPIs, has not yet been explored (Table 3).

Hopefully, these will be fully evaluated also in Europe and North America (vonoprazan has recently been approved by the US FDA), where the choice of antisecretory treatments remains limited. Only after worldwide extensive use can a critical evaluation of a new agent (especially belonging to a new drug class) be made, allowing clinicians to determine whether it is effective and safe and whether it is really superior to currently available treatments. Together with efficacy, also safety data are accumulating, in particular for vonoprazan, now in clinical use for more than 9 years. In a comprehensive systematic review of 77 studies [188], the incidences of any adverse effects AEs, drug-related AEs, serious AEs and AEs leading to drug discontinuation were not significantly different between patients taking vonoprazan and PPIs. However, since the incidence of AEs was higher in patients taking long-term use of vonoprazan than those taking short-term use of vonoprazan [188], data from studies from patients taking the drug from maintenance of reflux esophagitis are awaited with interest. However, the data from the VISION trial [189] concerning patients treated for 260 weeks with vonoprazan are reassuring.

As with every new drug, overuse and misuse can occur and can be avoided only with responsible marketing and thoughtful prescribing, together with careful monitoring of patients treated. At the present time, the indications for treatment with P-CABs should be for the difficult to treat acid-related disorders and unmet needs, where the benefit to risk is expected to be most favorable [15, 17].

## Data Availability

No datasets were generated or analysed during the current study.
